# ﻿*Sinosenecioyangii* (Asteraceae), a new species from Guizhou, China

**DOI:** 10.3897/phytokeys.210.89480

**Published:** 2022-09-27

**Authors:** Jing-Yi Peng, Dai-Gui Zhang, Tao Deng, Xian-Han Huang, Jun-Tong Chen, Ying Meng, Yi Wang, Qiang Zhou

**Affiliations:** 1 College of Biology and Environmental Sciences, Jishou University, Jishou 416000, Hunan, China; 2 Key Laboratory of Plant Resources Conservation and Utilization, Jishou University, College of Hunan Province, Jishou, Hunan 416000, China; 3 CAS Key Laboratory for Plant Diversity and Biogeography of East Asia, Kunming Institute of Botany, Chinese Academy of Sciences, Kunming 650201, Yunnan, China

**Keywords:** molecular evidence, morphology, pappus

## Abstract

A new species *Sinosenecioyangii* D.G. Zhang & Q. Zhou (Asteraceae, Senecioneae) from Guizhou Province, China, is described and illustrated based on its morphological characteristics and molecular evidence. It closely resembles *S.confervifer* and *S.guangxiensis*, the former in the scapigerous habit and smooth and glabrous achene surface, the latter in the calyculate involucre and purple abaxial leaf surface, and both in the shape and indumentum of leaf lamina, but differs markedly from the latter two in having fewer capitula and epappose achenes. Phylogenetic analysis based on nrITS and *ndhC-trnV* sequences shows that this new species belongs to the *S.latouchei* clade and is sister to *S.guangxiensis* with moderate support.

## ﻿Introduction

*Sinosenecio* B. Nordenstam (1978) (Senecioneae, Asteraceae) contains 45 species mainly distributed in central and southwestern parts of China (Chen et al. 2011; Liu and Yang 2012; Liu et al. 2019; Zou et al. 2020; Chen et al. 2022). This genus is characterized by subscapiform or leafy stems, palmately or rarely pinnately veined leaf lamina, solitary to numerous capitula, and ecalyculate or sometimes calyculate involucres, etc. (Jeffrey and Chen 1984). *Sinosenecio* encompasses two species assemblages, i.e. the *Sinosenecios.s.* group and the *S.oldhamianus* group, with different chromosome number (*x* = 30 vs. 24 or 13), patterns of endothecial cell wall thickenings (strictly polarized vs. polarized and radial), and phylogenetic affiliation (subtrib. Tussilagininae*s.s.* vs. subtrib. Thephroseridinae) (Liu 2010; Liu and Yang 2011a, b; Gong et al. 2016). These two groups also differ in geographical distribution. The former is restricted to mountainous regions around Sichuan Basin, southwestern China, and the latter is widely distributed in central and southern China, with two species extending to Indochina (Gong et al. 2016). However, a formal taxonomic adjustment is not yet proposed as phylogenetic relationships in subtrib. Thephroseridinae need to be further clarified (Nordenstam and Pelser 2011).

Libo County (Guizhou Province, China) belongs to the slope zone of transition from Guizhou Plateau to Guangxi Hilly Basin with typical karst topography and complex and diverse ecological environment (Tan 2010). In the past few years, some new species have been reported in this area, such as *Strobilantheshongii* (Chen et al. 2019) and *Petrocodonluteoflorus* (Fan et al. 2020). During our field investigation at Lihua Town, Libo County in March 2021, we found several unusual *Sinosenecio* populations that morphologically resemble two members of the *S.oldhamianus* group, namely *S.confervifer* (H. Léveillé) Y. Liu & Q. E. Yang and *S.guangxiensis* C. Jeffrey & Y. L. Chen, but differs markedly from them in several morphological features, respectively. After examining herbarium specimens and relevant literature, we verified that it represents an undescribed species. Here, we described it as *S.yangii* D. G. Zhang & Q. Zhou with report on its chromosome number and phylogenetic position.

## ﻿Materials and methods

### ﻿Morphological observation

Morphological examination and comparison of the new species with *S.confervifer* and *S.guangxiensis* were based on fresh materials and herbarium specimens. Chromosome observation was conducted according to Meng et al. (2010).

### ﻿Molecular analyses

To test the phylogenetic affiliation of *S.yangii*, we carried out phylogenetic analysis based on combined matrix of ITS and *ndhC-trnV* sequences. The matrix contained 23 accessions from 20 species, including the new species, 16 species of *S.oldhamianus* group, two of *Nemosenecio*, and an outgroup *Tephroserisflammea* (Turcz. ex DC.) Holub. The ITS and *ndhC-trnV* of *S.yangii* were sequenced in this study and the rest were downloaded from GenBank. The GenBank accession numbers are listed in Appendix 1. Total DNA was extracted from dried leaves using Plant Genomic DNA Kit DP305 (Beijing, China) and used as the template for polymerase chain reaction (PCR). The primers used in this study are listed in Table 1. Sequences obtained were edited using Sequencher-5.4.5 and then combined by Sequence Matrix-1.9 (Vaidya et al. 2011). Multi-sequence alignment and manual adjustment were conducted using programme CLUSTAL_W in Mega-X64 (Rédei 2008) and gaps were treated as missing data.

**Table 1.** Primers used in this study.

Region	Name	Primer sequence (5´–3´)
*nrITS*	ITS1	AGAAGTCGTAACAAGGTTTCCGTAGG
	ITS4	TCCTCCGCTTATTGATATGC
*ndhC-trnV*	ndhCretF	AAGTTTCTCCGGTCCTTTGC
	trnVretR	TCTACGGTTCGAGTCCGTATAG

Phylogenetic trees were constructed using Bayesian Inference (BI) and Maximum Likelihood (ML) in CIPRES Portal (https://www.phylo.org/portal2). BI and ML analyses were performed using MrBayes version-3.2 (Ronquist et al. 2012) and RAxML-8.2.10 (Stamatakis 2014), respectively. For BI analysis, GTR+G was selected as best-fitting model using Akaike information criterion (AIC) in JmodelTest 2-2.1.6 (Posada 2008). The Markov chain Monte Carlo analyses were run with four simultaneous chains of 10,000,000 generations sampling one tree every 1,000 generations. After the first 25% of trees were discarded as burn-in, the remaining trees were used to construct a majority-rule consensus tree with Bayesian posterior probabilities. ML analysis was performed with GTRCAT model, support values was calculated with 1,000 bootstrap replicates using a fast bootstrapping algorithm (Stamatakis et al. 2008).

## ﻿Results

### ﻿Morphology and taxonomy

**Figure 1.**
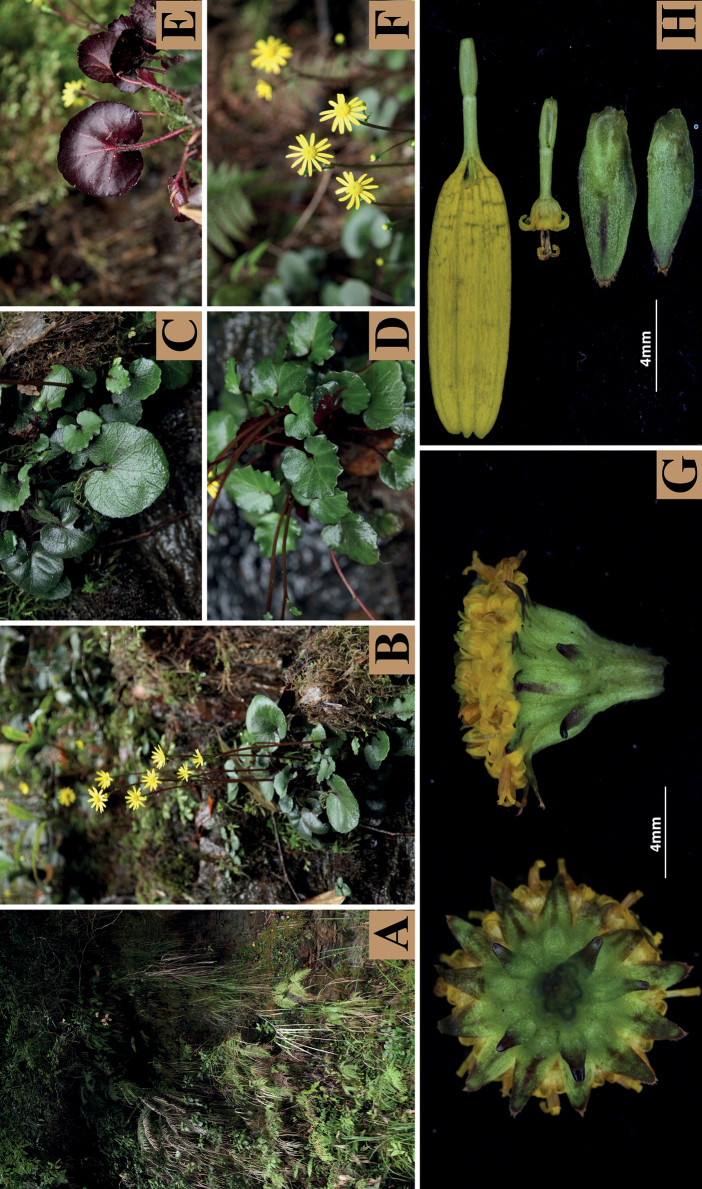
*Sinosenecioyangii***A** habitat **B** habit **C–E** leaves **F** capitulum **G** bottom and side of involucres (from left to right) **H** ray floret, disc floret and phyllary (from top to bottom).

**Figure 2.**
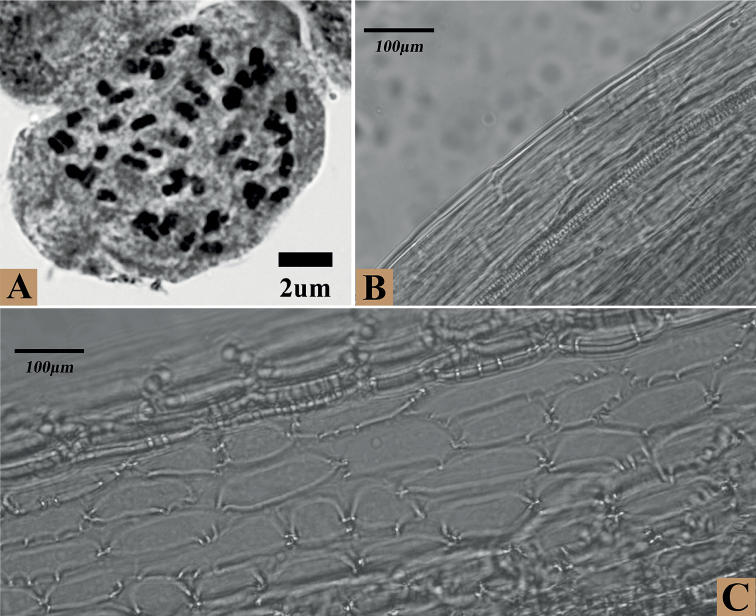
*Sinosenecioyangii***A** metaphase chromosomes (2*x* = 48) **B** smooth and glabrous achene surface **C** polarized and radial endothecial cell wall thickenings.

Morphological observation (Fig. 1) showed that *S.yangii*, *S.confervifer*, and *S.guangxiensis* share obvious resemblance in the leaf blade shallowly undulate and suborbicular, adaxially densely to sparsely villous and abaxially sparsely pubescent or nearly glabrous (Table 2). In addition, *S.yangii* is similar to *S.confervifer* in the stem leafless or with 1–2 bract-like leaf and smooth achene surface, and to *S.guangxiensis* in the calyculate involucre. Nevertheless, *S.yangii* differs from both species in having fewer capitula (usually 1–3) and epappose achenes. The metaphase chromosomes of this species were counted to be 2n = 48 (Fig. 2A). The achene surface was glabrous and smooth (Fig. 2B) and the anther endothecial cell wall thickenings were polarized and radial (Fig. 2C).

**Table 2.** Comparison of morphological characteristics among *Sinosenecioyangii*, *S.guangxiensis* and *S.confervifer*.

	*S.yangii*	*S.guangxiensis*	*S.confervifer*
Height (cm)	15–25	10–30	10–65
Leaf shape	Suborbicular or reniform, margin irregularly deltoid or rounded dentate, shallowly undulate or nearly entire	Suborbicular or reniform, margin coarsely repand or dentate with ovate-deltoid teeth	Orbicular or suborbicular, margin repand or lobed, with rounded or broadly deltoid mucronulate or obscurely mucronulate shallow teeth or lobes
Leaf size (cm)	2.5–4.5 × 2.5–6.5	2–6 × 2.5–7	1.5–6 × 2–6
Adaxial surface of leaf lamina	Green, densely or sparsely pubescent	Green or dark green, sparsely todensely villous or glabrous	Lustrous, green or deep green, densely or sparsely villous or glabrous
Abaxial surface of leaf lamina	Pale green or purplish red, sparsely arachnoid or nearly glabrous	Deep purplish red, densely white tomentose, sparsely villous or glabrescent	Pale green or slightly purple with sparsely arachnoid, veins villous or pubescent
Cauline leaves	1–2, bract-like	1–5, similar to radical ones	1–2, bract-like
Petiole base of cauline leaves	Expanded, not auriculate	Slightly expanded, not auriculate	Expanded, not auriculate
Number of capitula	Usually1, sometimes 2 or 3	2–7 or more, rarely 1	1–7 (–10) or more
Involucre	Calyculate	Calyculate	Not calyculate
Phyllaries	13	13	13
Chromosome number 2*x*	48	48	48
Achene surface	Smooth, glabrous	Papillate, pubescent	Smooth, glabrous
Pappus	Absent	Present	Present
Geographical distribution	Guizhou	Guangxi, southwestern Hunan	Hunan, Sichuan, Chongqing, Guizhou, Yunnan

### ﻿Phylogenetic analyses

**Figure 3.**
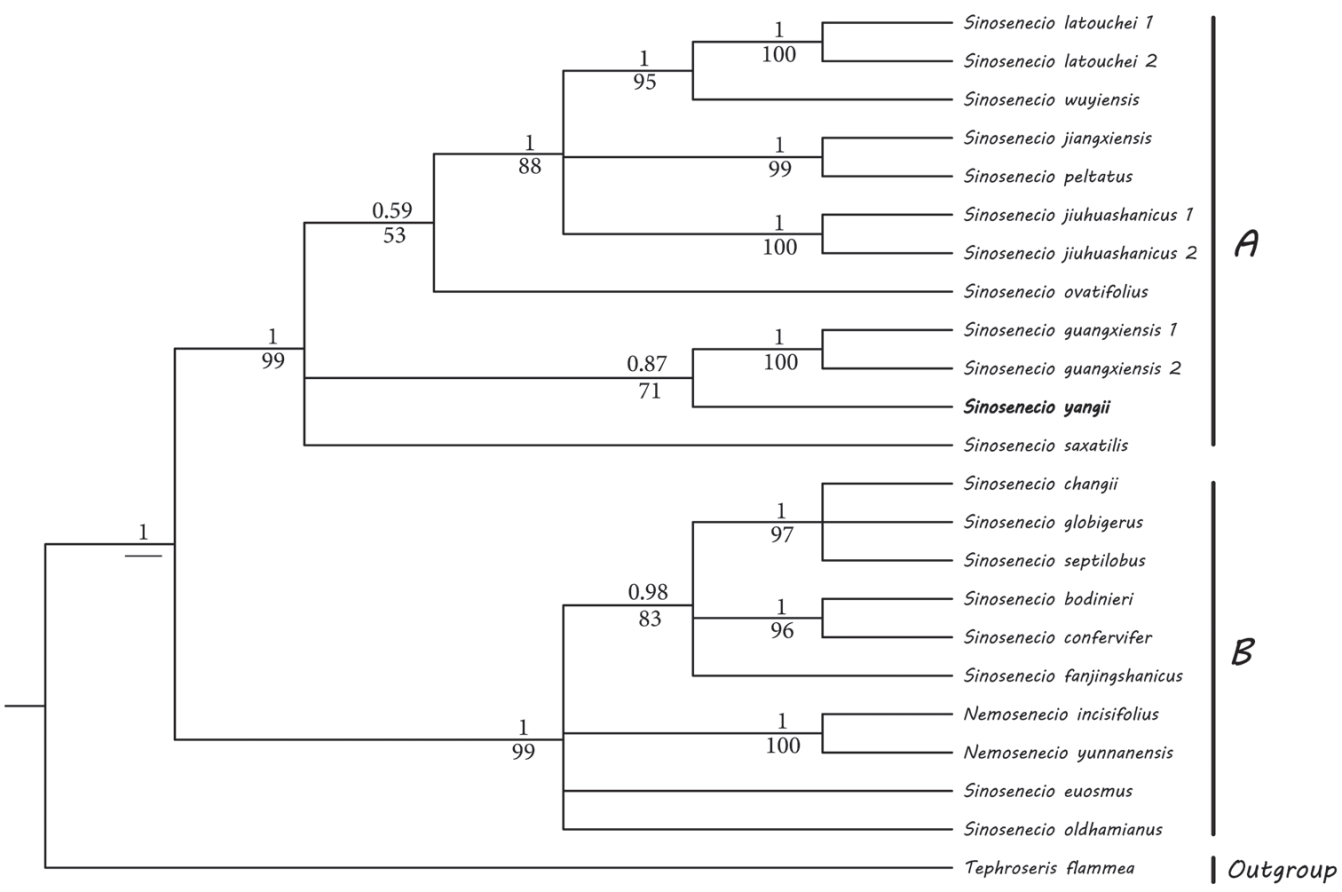
Bayesian phylogenetic tree based on the combined data of ITS and *ndhC-trnV* sequences. Numbers above and below branches are Bayesian posterior probabilities and ML bootstrap values, respectively. A and B represent the *S.latouchei* clade and *S.oldhamianus*-*Nemosenecio* clade. *Sinosenecioyangii* is noted in bold.

The combined matrix of ITS and *ndhC-trnV* sequences contained 1,324 aligned bp. Bayesian (BI) and Maximum likelihood (ML) trees had similar topologies. The BI tree was presented in Fig. 3 with BI posterior probability (BP) and ML bootstrap support values (LP) labelled on the branches. Ingroups were resolved into two clades, viz. the *S.latouchei* clade (BP = 1, LP = 99) and the *S.oldhamianus*-*Nemosenecio* clade (BP = 1, LP = 99). *Sinosenecioyangii* was resolved as sister to *S.guangxiensis* (BP = 0.87, LP = 71) in the former clade, while *S.confervifer* was recovered as a member in the latter clade.

## ﻿Discussion

Several lines of evidence demonstrated that *S.yangii* is a member of the *S.oldhamianus* group. *Sinosenecioyangii* has a base chromosome number of x = 24 (Fig. 2A) and polarized and radial anther endothecial cell wall thickenings (Fig. 2C), which are typical of the *S.oldhaminanus* group. Analyses of ITS and *ndhC-trnV* sequences also corroborated its phylogenetic affiliation, resolving it as sister to *S.guangxiensis* in the *S.latouchei* clade of *S.oldhaminanus* group.

*Sinosenecioyangii* was morphologically and phylogenetically close to *S.guangxiensis* in the *S.latouchei* clade. However, there are differences in morphology, distribution and ecology between the two species. *S.yangii* is easily distinguished from *S.guangxiensis* in the stem leafless or with 1–2 bract-like leaf (vs. 1–5 cauline leaves), fewer capitula (vs. 2–7 or more), smooth and glabrous achene surface (vs. papillate and pubescent), and epappose achenes (vs. present pappus). From the perspective of distribution area, the former is restricted to Libo County in Guizhou, appearing on the wet rock cliff, and the geographical location is adjacent to the border with Guangxi province. The latter is distributed in the Guangxi and southwestern Hunan, growing on the damp, shady places or rocky places at mountain summits. To some extent, the close relationship between these two species may also be related to their distributional ranges adjacent to each other. Additionally, it is worth noting that the epappose achenes of *S.yangii* is a character previously never recorded in the *S.latouchei* clade.

### ﻿Taxonomic treatment

#### ﻿ Sinosenecio yangii

Taxon classificationPlantaeAsteralesAsteraceae

D. G. Zhang & Q. Zhou sp. nov.

AF64558E-1298-5967-AE6E-AA5C7E0AFAE1

urn:lsid:ipni.org:names:77305747-1

Figs 4 , 5

##### Type.

China. Guizhou: Libo County, Lihua Town, 25°36'53"N, 108°12'63"E, on rock cliff by the side of a rural road, elev. 347 m, 16 March 2021, *D. G. Zhang & T. Deng* 14231. (holotype: JIU! ; isotype: JIU!).

##### Description.

Scapigerous herbs. Rhizomes short and stout with many fibrous roots. Stems slender, scapiform, erect or declining, solitary or several, 13–22 cm long, basally reddish-brown and sparsely white villous, almost smooth in upper part. Radical leaves several; petiole ca. 3–6.5 cm long, densely villous or glabrescent, basally expanded, not auriculate; lamina suborbicular or reniform, ca. 2.5–4.5 × 2.5–6.5 cm, base cordate, margin irregularly triangular dentate, shallowly undulate or entire, apex slightly acute; adaxially green, densely or sparsely pubescent, abaxially pale green or purplish red, sparsely arachnoid or nearly glabrous. Upper leaves 1 or 2, bract-like, shortly petiolate, lanceolate. Capitula usually 1–3, peduncles slender, ca. 2–3.5 cm long, with a basal linear bracteole, or with 1–2 small linear bracteoles in the upper part. Involucres campanulate, calyculate with 2–3 bracteoles or more; phyllaries ca. 13, lanceolate, ca. 6 mm long, with ciliate margin, apically acute or obtuse and sometimes purplish. Ray florets ca. 13, corolla tube 3 mm long, glabrous; ray yellow, oblong, ca. 12 mm long, 4-veined, apically 3-denticulate. Disc florets numerous; corolla yellow, 4 mm, with ca. 1.5 mm glabrous tube and 0.85 mm limb. Anthers oblong, 5, ca. 1.2 mm long, basally obtuse. Style branches ca. 0.5 mm long, puberulent. Achenes ca. 1 mm long, smooth and glabrous. Pappus absent.

**Figure 4.**
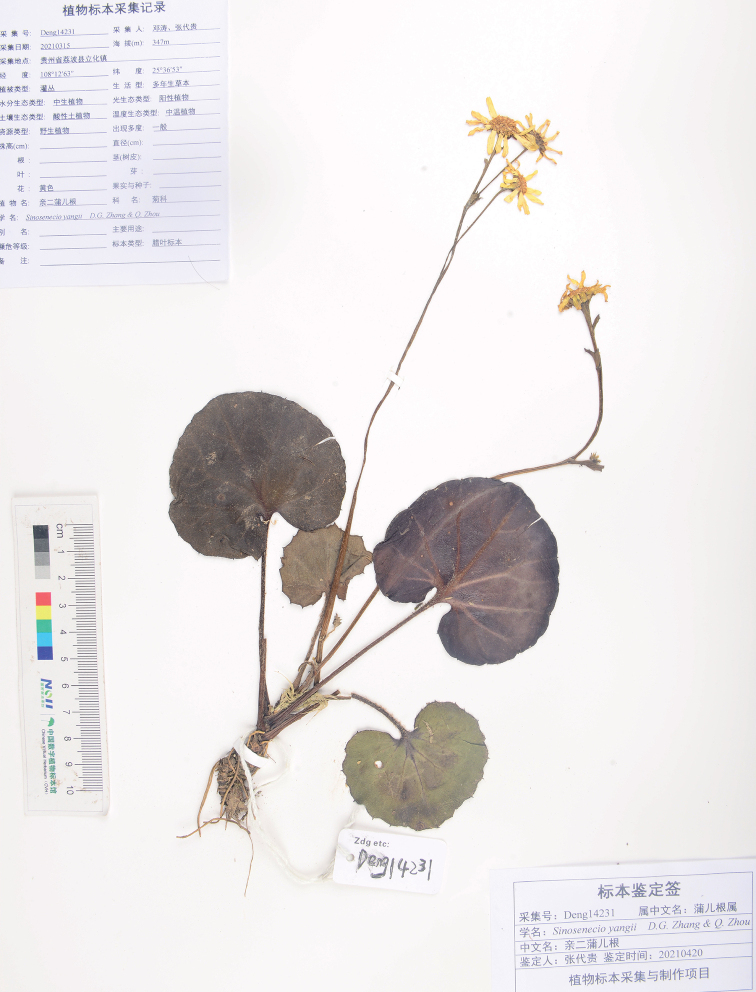
Holotype sheet of *Sinosenecioyangii* D. G. Zhang & Q. Zhou.

**Figure 5.**
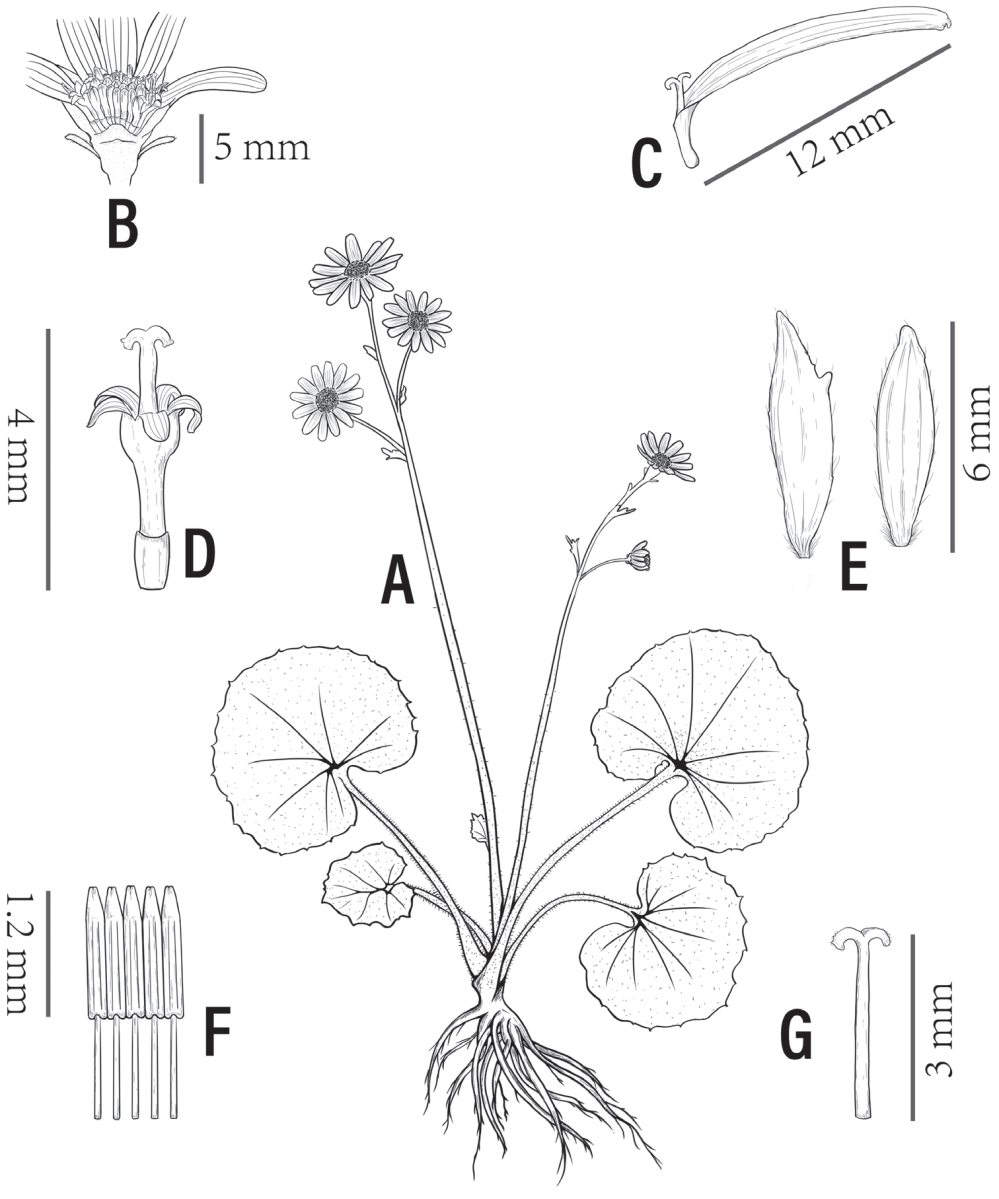
*Sinosenecioyangii***A** habit **B** capitulum **C** ray floret **D** disk floret **E** phyllary **F** stamens **G** style (drawing by Chu-miao Xie).

##### Phenology.

Flowering from March to May, fruiting from April to June.

##### Etymology.

The species was named after Professor Qin-er Yang, an expert in the field of *Asteraceae* at the Chinese Academy of Sciences. The Chinese name is given as “亲二蒲儿根” (qīn èr pú ér gēn).

##### Distribution and habitat.

**Figure 6.**
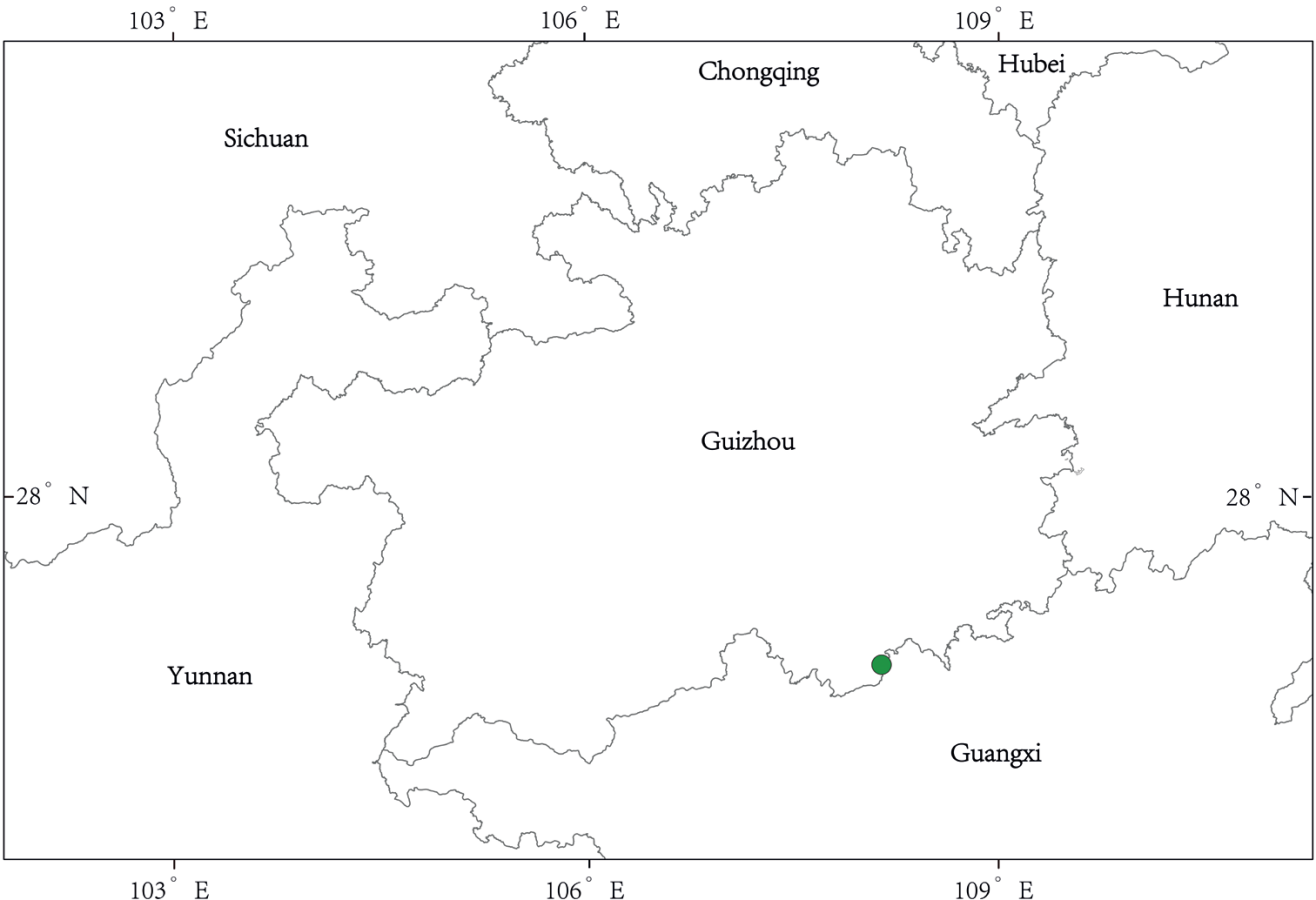
Distribution of *Sinosenecioyangii*.

*Sinosenecioyangii* is known from Lihua Town, Libo County, Guizhou Province, China (Fig. 6). It was collected from a rock cliff by the side of a rural road in this town, at an altitude of 347 m.

### ﻿Key to species of the *S.latouchei* clade

**Table:** 

1	Pappus absent	***S.yangii***
–	Pappus present.	**2**
2	Leaf lamina peltate	***S.peltatus***
–	Leaf lamina not peltate	**3**
3	Involucres calyculate	**4**
–	Involucres ecalyculate.	**5**
4	Cauline leaf absent or 1 and bract-like; base of petiole of cauline leaf slightly auriculate; capitula solitary, rarely 2 or 3	***S.jiangxiensis***
–	Cauline leaves 1–5, similar to radical ones; base of petiole of cauline leaves never auriculate; capitula 1–5 or more	***S.guangxiensis***
5	Ovaries and achenes glabrous	**6**
–	Ovaries and achenes pubescent.	**7**
6	Leaf lamina broadly flabellate or suborbicular, dentate or palmately lobed to 1/2, lobes apically 2 or 3-denticulate, both surfaces glabrous	***S.wuyiensis***
–	Leaf lamina reniform or suborbicular, regularly 5–7-palmatilobed, lobes ovate-triangular, both surfaces glabrous or sometimes white tomentose abaxially and later glabrescent	***S.saxatilis***
–	Leaf lamina ovate, broadly ovate, rarely ovate-orbicular, inconspicuously undulate-dentate, adaxial surface villous, sometimes sparsely arachnoid, and abaxial surface villous and densely white arachnoid	***S.ovatifolius***
7	Stem erect or flexuous; cauline leaves 1–3; leaf lamina adaxially villous with spreading hairs; leaf auricles 4–10 mm in diameter	***S.latouchei***
–	Stem erect; cauline leaves 3–7; leaf lamina adaxially pubescent with appressed hairs or sparsely or densely white tomentose; leaf auricles 7–30 mm in diameter	***S.jiuhuashanicus***

## Supplementary Material

XML Treatment for Sinosenecio yangii

## ﻿Appendix 1

**Table A1.** GenBank accessions of species used in this study.

Species	GenBank accession number of ITS / *ndhC-trnV*
*Tephroserisflammea*	KU696137 / KU750769
*Nemosenecioyunnanensis*	KU696047 / KU750695
*Nemosenecioincisifolius*	KU696045 / KU750694
*Sinoseneciolatouchei*	JQ797428 / KU750748
*Sinoseneciolatouchei*	JQ797429 / KU750749
*Sinoseneciowuyiensis*	JQ797431 / KU750764
*Sinoseneciojiangxiensis*	KT149879 / KU750743
*Sinoseneciopeltatus*	MK818500 / –
*Sinoseneciojiuhuashanicus*	JQ797426 / KU750746
*Sinoseneciojiuhuashanicus*	JQ797425 / KU750745
*Sinosenecioovatifolius*	MT522620 / –
*Sinosenecioguangxiensis*	JQ797432 / KU750738
*Sinosenecioguangxiensis*	JF978599 / KU750739
*Sinosenecioyangii*	OM413747 / OM371331
*Sinoseneciosaxatilis*	JQ797430 / KU750757
*Sinoseneciochangii*	AY176164 / KU750721
*Sinosenecioglobigerus*	AY176159 / KU750736
*Sinosenecioseptilobus*	AY176161 / KU750758
*Sinoseneciobodinieri*	KT149888 / KU750720
*Sinosenecioconfervifer*	KT149891 / KU750723
*Sinoseneciofanjingshanicus*	KT149886 / KU750732
*Sinosenecioeuosmus*	JF978589 / KU750730
*Sinoseneciooldhamianus*	JF978616 / KU750753
